# Robust ultra-low-friction state of graphene via moiré superlattice confinement

**DOI:** 10.1038/ncomms13204

**Published:** 2016-10-19

**Authors:** Xiaohu Zheng, Lei Gao, Quanzhou Yao, Qunyang Li, Miao Zhang, Xiaoming Xie, Shan Qiao, Gang Wang, Tianbao Ma, Zengfeng Di, Jianbin Luo, Xi Wang

**Affiliations:** 1 State Key Laboratory of Functional Materials for Informatics, Shanghai Institute of Microsystem and Information Technology, Chinese Academy of Sciences, 865 Changning Road, Shanghai 200050, China; 2 State Key Laboratory of Tribology, Tsinghua University, Beijing 100084, China; 3 AML, CNMM, Department of Engineering Mechanics, Tsinghua University, Beijing 100084, China

## Abstract

Two-dimensional (2D) materials possess outstanding lubrication property with their thicknesses down to a few atomic layers, but they are easily susceptible to sliding induced degradation or ubiquitous chemical modification. Maintaining the superior lubricating performance of 2D materials in a harsh working environment is highly desirable yet grandly challenging. Here we show that by proper alignment of graphene on a Ge(111) substrate, friction of graphene could be well preserved at an ultra-low level even after fluorination or oxidation. This behaviour is experimentally found to be closely related to the suppression of molecular-level deformation of graphene within the moiré superlattice structure. Atomistic simulations reveal that the formation of an interconnected meshwork with enhanced interfacial charge density imposes a strong anchoring effect on graphene even under chemical modification. Modulating molecular-level deformation by interfacial confinements may offer a unique strategy for tuning the mechanical or even chemical properties of 2D materials.

## Abstract (web summary)

Two-dimensional materials show remarkable lubrication properties, yet chemical modifications may hinder such capabilities. Here, the authors show that when graphene is aligned on a Ge(111) substrate, ultra-low friction can be preserved even after graphene fluorination or oxidation.

## Graphical abstract

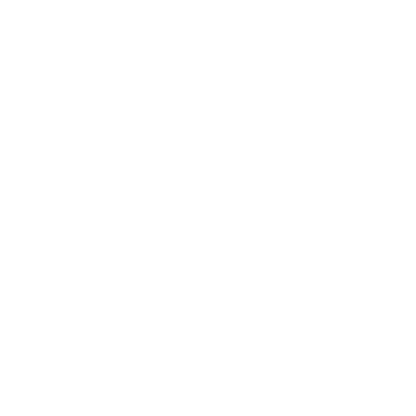

Friction exists almost in every mechanical system with moving parts, and it becomes more prominent for small-scale devices due to their high surface to volume ratio. For many applications, it is often desirable to control and, in many cases, to minimize friction^1^. Unfortunately, lubricating solid contacts robustly remains a long-standing challenge especially at the micro- and nanoscale^1^,^2^. Recently, 2D materials have attracted much attention as they still exhibit superior lubrication property even with only a few atomic layers^3^,^4^,^5^. Despite the great potential as atomically thin lubricants, the lubrication performance of these 2D materials is easily deteriorated when subjected to chemical modifications stemming from the working environments or contact sliding^6^,^7^,^8^,^9^,^10^. For example, oxidation^8^, hydrogenation^8^ or fluorination^9^,^10^ from the external circumstance can easily lead to the dramatically increased friction of 2D materials. Preservation of ultra-low-friction state of 2D materials in various environments has become necessary and essential for practical tribological applications. Previous study has suggested that adhesive interaction between 2D materials and their substrates could influence the contact configuration via the puckering effect, thereby affecting friction^4^,^11^. In particular, it has shown that, compared with a weakly adherent substrate (silicon oxide), a highly adhesive and ultra-flat mica substrate can significantly reduce friction of graphene by suppressing the laminar-level puckering effect^4^.

In this work, we demonstrate that the moiré superlattice formed between graphene and germanium can impose a strong anchoring to cement the graphene to Ge substrate at the atomic scale. The interfacial confinement effectively suppresses the molecular-level deformation of graphene, and preserves the ultra-low-friction state even when the surface is actively fluorinated or oxidized. Therefore, our study suggests that the mechanical or even chemical properties of 2D materials can be tuned through interfacial confinements by modulating molecular-level deformation of 2D materials.

## Results

### Preservation of ultra-low-friction state on fluorinated graphene

**Figure 1:**
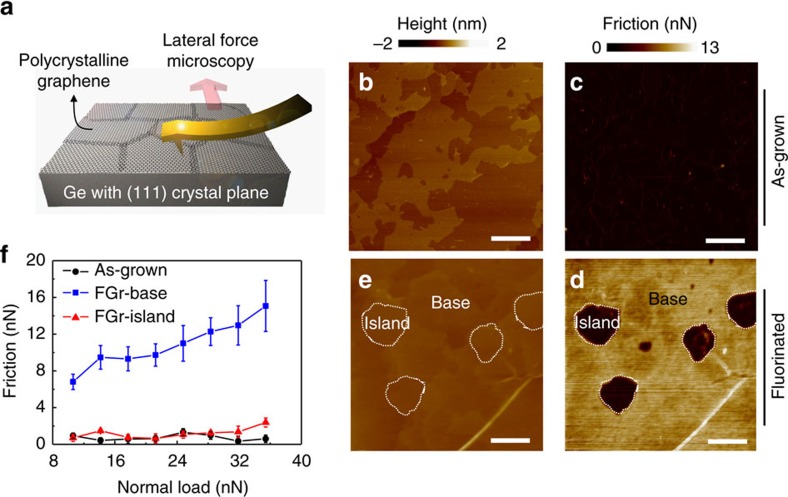
Experimental setup and microscale friction measurement on as-grown graphene and FGr. (**a**) A schematic diagram of the experimental setup. Topography and friction force images acquired simultaneously on surface of (**b**,**c**) as-grown graphene/Ge(111) and (**d**,**e**) FGr/Ge(111). Scale bars, 200 nm (**b**–**e**). (**f**) Plot of friction force versus normal load measured on the as-grown and FGr samples, error bars represent the s.d. of measurements at the given normal load.

In this work, a continuous film of monolayer polycrystalline graphene with a weak domain orientation preference^12^ was grown on Ge(111) through chemical vapour deposition. After growth, the graphene was exposed to SF_6_ plasma and its top surface was fluorinated as confirmed by Raman and X-ray photoelectron spectroscopies (see Supplementary Figs 1 and 2 and Supplementary Note 1). Lateral (friction) force images were taken with an atomic force microscope at ambient conditions, as schematically shown in Fig. 1a. The topographic image of the as-grown graphene is dominated by the morphology of the underlying Ge(111) surface with clear atomic steps (Fig. 1b). Friction on as-grown graphene (Fig. 1c) is ultra-low and rather uniform with its magnitude close to the previous reports^4^. In contrast, friction on graphene after fluorination becomes inhomogeneous and a clear contrast is observed. More specifically, friction of most areas (denoted as base) is significantly increased (Fig. 1d,f), which has been commonly observed in chemically functionalized graphene^8^,^9^,^10^. However, there coexist some randomly distributed islands (denoted as island), where surface friction remains ultra-low and is comparable to that of as-grown graphene (Fig. 1d,f). These low-friction island regions have no obvious morphological contrast compared with those high-friction base regions (Fig. 1e). This preservation of ultra-low-friction state of graphene after energetic functionalization (for example, fluorination) has not been reported based on our knowledge. The low-friction state on islands is reproducible for all fluorinated graphene(FGr)/Ge(111) samples tested (see Supplementary Fig. 3). In addition, similar friction contrast between island and base is always observed for different scanning velocities or under various normal loads (see Supplementary Fig. 4).

**Figure 2:**
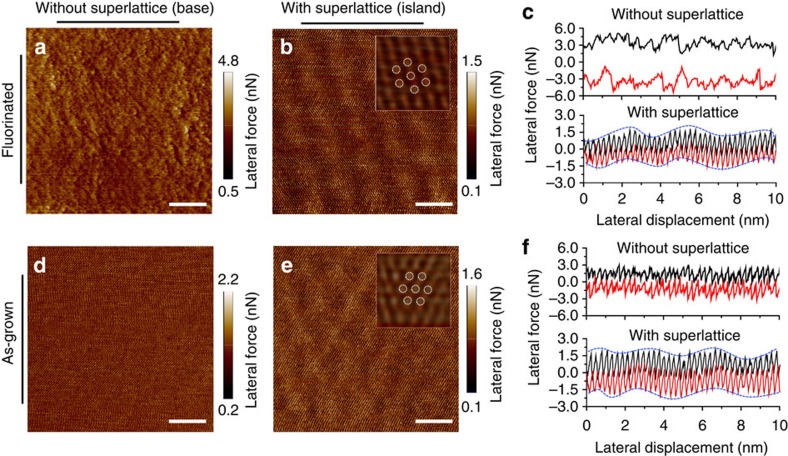
High-resolution lateral force images acquired from FGr and as-grown graphene. (**a**,**b**) Lateral force images (forward scan direction) from the base and the island regions of FGr, respectively. (**c**) Corresponding friction curves measured in **a**,**b**. A long-range undulation together with atomic stick-slip could be observed on **b**. (**d**,**e**) Lateral force images (forward scan direction) from two distinct regions of as-grown graphene. Only regular atomic stick-slip is observed on **d**, while both atomic stick-slip and an extra long-range undulation appear on **e**. (**f**) Corresponding lateral force curves measured in **d**,**e**. Fourier transform filtered images inserted in **b**,**e** show clear sixfold symmetric superlattice. Scale bars, 4 nm.

To explore the origin of the friction contrast on the FGr/Ge(111) heterostructure, we have taken detailed friction measurements across the base (high friction) and the island (low friction) regions, respectively. As shown in the lateral force image in Fig. 2a and the line traces in Fig. 2c, no regular atomic stick-slip can be observed in the high-friction base region suggesting a disordered nature of the surface^10^. However, in the island regions where the ultra-low-friction behaviour is preserved, atomic stick-slip can be clearly observed with a superposed long-range variation forming a sixfold symmetric superlattice structure, see Fig. 2b and the dotted trend lines in Fig. 2c. We also performed high-resolution friction measurements on as-grown graphene. Although clear stick-slip behaviour is always observed, the lateral force images on the as-grown graphene reveal two distinct types of regions: one without visible superlattice (Fig. 2d) and one with visible superlattice (Fig. 2e,f). Although the detailed mechanism for the long-range variation is less clear, it can originate from a periodically undulated energy corrugation^13^ or stiffness^14^. Intuitively, the contrast between Fig. 2a,b seems to associate with the presence of the superlattice structure.

### Moiré pattern and evolution of molecular structure revealed by STM

**Figure 3:**
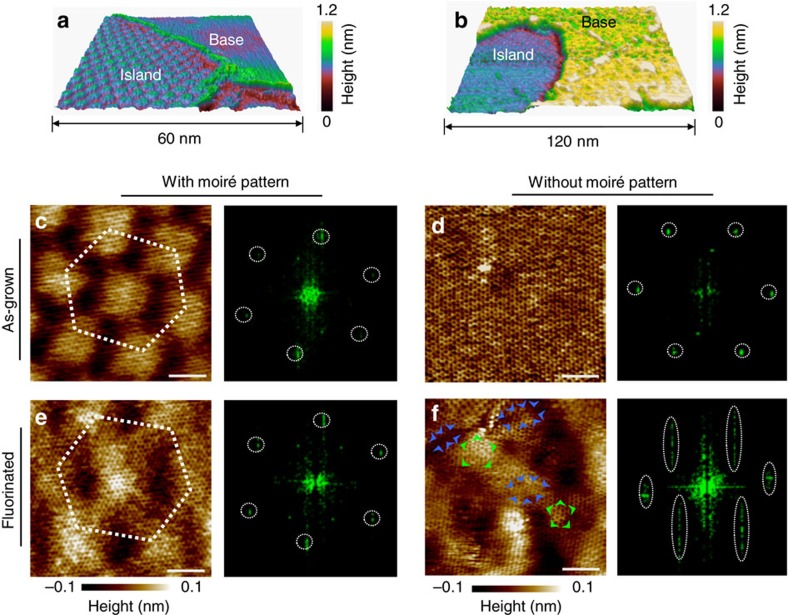
Surface morphology and lattice structure measured by STM. Representative 3D topographic images containing both base and island regions collected on (**a**) as-grown graphene and (**b**) FGr, respectively. (**c**,**d**) High-resolution topographic STM images acquired from the island and the base regions from **a**, together with the corresponding Fourier transform patterns. (**e**,**f**) High-resolution atomic lattice images acquired from the island and the base regions from **b**, together with the corresponding Fourier transform patterns. Although the outline of moiré pattern becomes slightly vague, the hexagonal lattice structure of graphene remains almost intact in the island region as indicated by the standard sixfold symmetry of the Fourier transform pattern **e**. The lattice structure is severely distorted in the base region, and blue and green arrows highlight the compressed and stretched lattices **f**, respectively. Scale bars are 2 nm. 3D, three-dimensional.

The impact of superlattice structure on friction was further investigated by scanning tunnelling microscopy (STM). The STM image from the island region (see Fig. 3a) indicates that the superlattice structure in the lateral force image (Fig. 2e) coincides with the moiré pattern formed between graphene and Ge(111). Moiré pattern often appears for single crystal surfaces with periodic overlayers^15^,^16^,^17^. Considering the lattice mismatch (38.5%) between graphene and Ge(111), the moiré pattern will have a periodical length of ∼4 nm when graphene is aligned on Ge(111). Because increasing relative rotation angle leads to rapid decrease of its periodical length, moiré patterns are only visible in STM images for regions where graphene and Ge(111) lattices are nearly aligned (see Supplementary Fig. 5 and Supplementary Note 2). Representative three dimensional (3D) STM topographic images collected at constant-current mode on both as-grown and fluorinated films are compared in Fig. 3a,b. Large out-of-plane deformation of the graphene surface can be clearly observed in the base regions of the fluorinated samples, while the morphology of the island regions with moiré pattern barely changes after fluorination (see more in Supplementary Fig. 6). The atomically resolved STM images of the base regions indicate that the molecular-level deformation occurs (comparing with as-grown graphene in Fig. 3d) with severe non-uniform lattice distortion and wrinkles or even ridges (Fig. 3f and the inset). In contrast, similar to the as-grown graphene (Fig. 3c), only negligible lattice distortion or wrinkling is found in the island regions with visible moiré pattern after fluorination (Fig. 3e). Extensive tribological studies^5^,^18^,^19^,^20^,^21^ have shown that sliding on a disordered and corrugated surface or interface usually leads to higher friction and more energy dissipation. Therefore, we speculate that the preservation of ultra-low-friction state may be attributed to less degree of fluorination or strongly suppressed molecular-level deformation of graphene in regions with a moiré pattern.

**Figure 4:**
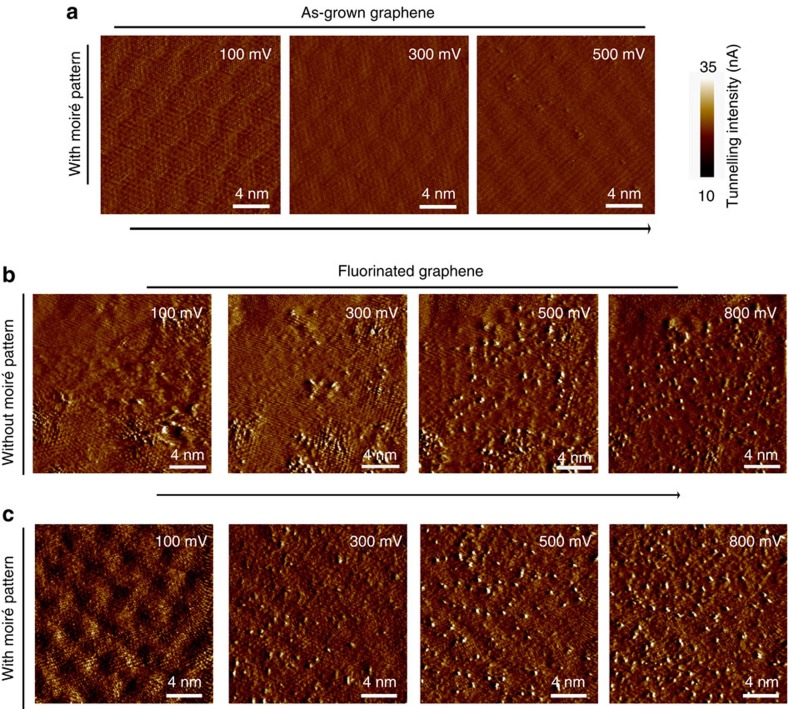
Sweeping voltage STM of as-grown and FGr. STM tunnelling current images recorded with varied scanning voltage in the regions (**a**) with moiré pattern of the as-grown graphene. (**b**,**c**) Acquired from the regions without and with moiré pattern of the FGr, respectively. The appearance of protrusions depends on the scanning voltage, indicating the co-existence of various C_*n*_F structures in the regions of FGr with and without moiré pattern.

Recent studies have reported that presence of moiré pattern may noticeably affect the self-assembly process of adatom clusters^22^ and change the catalytic activity on surfaces^23^. To check whether the less molecular deformation in regions with moiré pattern was due to less degree of fluorination, we performed further STM measurements to distinguish the fluorination levels for the fluorinated samples. Theoretical works have predicted that various C_*n*_F structures formed during fluorination process could open the energy bandgap of graphene^24^, resulting in local density of states with different energy levels. The tunnelling current at C_*n*_F structures with narrower energy bandgap can be induced by relatively low-bias voltages of STM, while high-bias voltages are required for the tunnelling of C_*n*_F structures with wider energy bandgap. For as-grown graphene on Ge(111), a moiré pattern is clearly visible by STM with sweeping bias voltages (100–500 mV) (Fig. 4a). However, the STM tunnelling current mapping of FGr (both with/without moiré pattern regions) under variable bias voltages *V*_b_ (100–800 mV) shows obvious tunnelling protrusions (increased tunnelling current) induced by fluorine attachment and the protrusion density increases markedly with increasing sample bias as show in Fig. 4b,c. From the comparison between Fig. 4b,c, it appears that the number of fluorination sites in the regions with a moiré pattern is not significantly different from that in the regions without a moiré pattern, although the distribution seems to be slightly more regular in the former case^22^,^25^. The STM results, together with adhesion measurements from regions with and without a moiré pattern (see Supplementary Figs 7 and 8 and Supplementary Note 3), essentially exclude different degree of fluorination as a primary mechanism for the observed friction contrast in the two regions.

## Discussion

**Figure 5:**
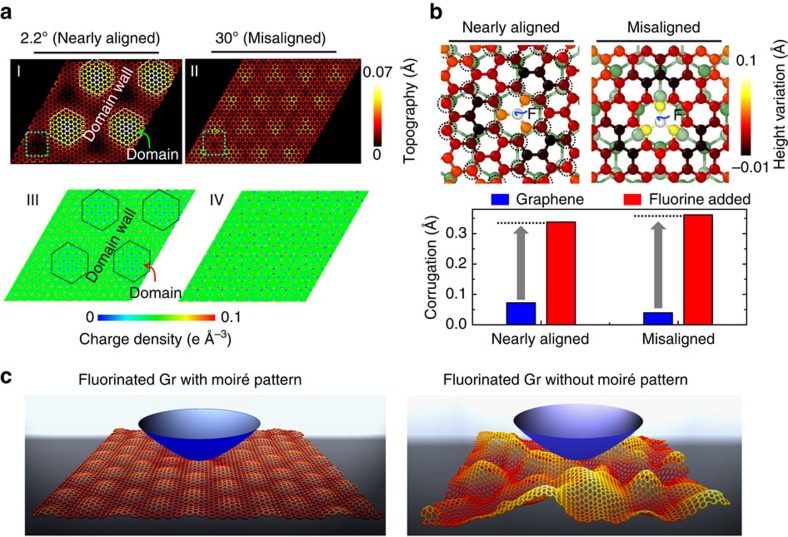
The anchoring effect revealed by the atomistic simulations. (**a**) DFT-relaxed morphology (I and II) of Gr/Ge(111) surface with rotation angles of 2.2° and 30°. Domain and domain wall can be distinguished in I. The corresponding interfacial charge density maps of I and II are shown in III and IV, respectively. (**b**) Atomic configuration (colour of top graphene layer atoms represents height change after fluorination) in the regions marked by the squares in 5a-I and 5a-II. The dotted circles highlight the projected overlaps between top C atoms and underneath Ge atoms. The lower panel presents the variation of graphene corrugation magnitude after the F atom is added. (**c**) Schematic representations showing the atomic configurations of backbone carbon atoms of graphene with/without moiré pattern after fluorination during friction measurement.

To investigate the possible confinement effect from the moiré pattern, density functional theory (DFT) calculations of graphene on Ge(111) substrate were performed. The relaxed morphology (Fig. 5a-I) shows a moiré pattern consisting of domain regions (peak) isolated by domain walls (valley) when a rotation angle of 2.2° (nearly aligned case with the periodical length of 3.2 nm, close to the experimental value) is imposed. The morphological undulation coincides with the periodic distribution (Fig. 5a-III) of interfacial charge density, which is an essential indication of the interaction strength between graphene and Ge substrate^26^. Consistent with the experimental observations (see Supplementary Fig. 9), the interfacial charge density is more enhanced in domain wall compared with that in the domain region. This enhanced interaction at the domain wall region is associated with the local interlayer stacking geometry: larger projected overlaps between C atoms of graphene and Ge atoms of substrate results in stronger interaction, as highlighted by the dotted circle in domain wall regions (Fig. 5b). In contrast, when the interface is highly misaligned at a large rotation angle, for example, 30°, the morphological undulation and moiré pattern becomes barely visible (Fig. 5a-II)^27^ and the corresponding charge density is sparsely distributed at the interface (Fig. 5a-IV). In the nearly aligned case, the interconnected domain wall regions with enhanced interfacial charge density spread collectively over the substrate, which acts as an extended meshwork to tightly anchor the graphene onto the substrate^26^,^28^. This unique anchoring effect becomes more evident when a fluorine atom is added to mimic the fluorination process. As shown in Fig. 5b, adding F atom tends to pull the neighbouring C atoms up due to the *sp*^2^–*sp*^3^ rehybridization. In the nearly aligned case, the local out-of-plane deformation is noticeably suppressed due to the anchoring effect; while the anchoring effect is attenuated in the misaligned case, resulting in a larger deformation (see lower panel of Fig. 5b). We have to note here that the periodic boundary condition for relatively small unit cell imposed in the DFT calculations and the limitation of adding single fluorine atom may underestimate the difference in deformation between the two cases. In real experiments, the molecular-level deformation of graphene lattice without moiré pattern can be much more pronounced than that with moiré pattern as exhibited by the STM images (Fig. 3e,f), which is schematically depicted in Fig. 5c. Previously, both experimental and theoretical studies have shown that disordered and defective surfaces usually exhibit larger friction compared with an ordered and defect-free surfaces, primarily due to the increase of the number and type of low-energy modes or stronger corrugation of the lateral potential^19^,^29^,^30^. Therefore, sliding on the misaligned regions will result in a much higher friction because of the disordered and the highly corrugated nature of the surface, while friction in the aligned regions could be much lower due to suppression of molecular-level deformation by the anchoring effect (Fig. 5c).

Different anchoring effect in the regions with and without moiré pattern might result in different dynamic responses when the graphene surface was slid by a tip. This in turn might cause different velocity dependence of friction on different regions. However, as shown in Supplementary Fig. 4, we did not see a clear variation trend of friction on sliding speed, which may be due to the relatively small domain size and/or high speeds adopted in our tests. In addition, because friction on as-grown samples was rather low with relatively large uncertainties, a more compliant/sensitive probe might also be needed to better distinguish the subtle variation in future study. Nevertheless, our work clearly demonstrates that the friction contrast between regions with and without a moiré pattern after graphene fluorination was always high and did not change qualitatively with the tip scanning speed.

The anchoring effect, which originates from the interconnected domain wall meshwork of the moiré pattern in the graphene/Ge(111) heterostructure, may be generally applied to preserve the ultra-low-friction state of graphene when utilized in other chemically aggressive environments. This was indeed confirmed by the friction behaviour of oxidized graphene/Ge(111) structure: the regions with moiré superlattice did preserve ultra-low-friction state of graphene as well (see Supplementary Figs 10 and 11 and Supplementary Note 4). Therefore, the formation of moiré superlattice between 2D material and a substrate is an effective strategy to maintain the superior lubrication property of 2D material in a harsh chemical environment. With recent advances in synthesis of large-scale 2D films with precisely controlled interfacial structures for various materials^27^,^31^,^32^,^33^,^34^, our finding potentially opens a unique route of tuning the nanomechanical properties of 2D materials systems for a wide variety of applications.

## Methods

### Samples preparation

P-type Ge(111) (175 μm thick, AXT) substrates with resistivity of 0.01–0.05 Ohm cm were used in the experiments. The graphene film was synthesized by chemical vapour deposition in a horizontal tube furnace with H_2_: CH_4_: Ar=0.73: 23: 230 s.c.c.m. at the growth temperature of 916 °C for 300 min. Fluorination of graphene was performed by exposing the sample into the SF_6_ plasma for 3 min in the OXFORD Plasma Lab system-100. To suppress the effect of ion bombardment to graphene, the sample was placed ‘face down' (graphene faced towards the bottom electrode) using a quartz holder. The SF_6_ flow rate was set as 40 s.c.c.m. and the plasma power was 50 W (bias voltage ∼23 V). During fluorination, the chamber pressure and temperature of the sample stage were 96 mTorr and 60 °C, respectively.

### Samples measurements

Bruker Multimode 8 system was applied to perform the friction, height, pull-off force measurements and STM at ambient conditions (temperature ∼22 °C, relative humidity ∼30%). During lateral force microscopy measurements, silicon probes with nominal tip radius of curvature ∼2 nm (SNL-10, Bruker) were chosen. Deflection sensitivity of the probe is 33.66 nm V^−1^ and the spring constant at the room temperature is 0.35 N m^−1^. Lateral force microscopy images were obtained simultaneously with the topographic images. The friction force was calculated by subtracting the forward and backward friction signals on each scan line and divided by two. The normal and lateral force were calibrated using the ramp mode^35^ and the diamagnetic levitation spring system^36^. High-resolution lateral force images in Fig. 2 in the main text were acquired with an atomic force microscope (NT-MDT spectrum Instruments) in a dry N_2_ environment with a Si tip (CSC37, MikroMasch). STM images were acquired with a platinum–iridium (Pt–Ir) alloy tip in normal ambient under the constant-current mode.

### First-principles calculations

The DFT calculations with a non-local optB86b-vdW exchange-correlation functional^37^,^38^ were performed using the Vienna *ab initio* Simulation Package^39^. The projector-augmented-wave method was utilized to model the core electrons. The energy cutoff of the expanded wave functions was set to 400 eV. A 32 × 32 × 30 Å slab supercell containing three-layer Ge(111) atoms and monolayer graphene with a vacuum gap of more than 19 Å was used. The rotation angles between graphene and Ge(111) substrate were selected as 2.2° and 30°. The graphene and top Ge(111) layer were allowed to relax until the forces on all the relaxed atoms were less than 0.02 eV Å^−1^. All the calculations were done using experimental Ge lattice constant (*a*=5.6575 Å).

### Data availability

The data that support the findings of this study are available from the corresponding authors on request.

## Additional information

**How to cite this article:** Zheng, X. *et al*. Robust ultra-low-friction state of graphene via moiré superlattice confinement. *Nat. Commun.* **7,** 13204 doi: 10.1038/ncomms13204 (2016).

## Supplementary Material

Supplementary Information Supplementary Figures 1-11, Supplementary Notes 1-4 and Supplementary References
