# Supplementary material for: Robust ultra-low-friction state of graphene via moiré superlattice confinement
Source: Nat Commun. 2016 Oct 19;7:13204. doi: 10.1038/ncomms13204 (PMC5075778; doi:10.1038/ncomms13204)
Supplement: Supplementary Information — Supplementary Figures 1-11, Supplementary Notes 1-4 and Supplementary References [file ncomms13204-s1.pdf]

Supplementary Information

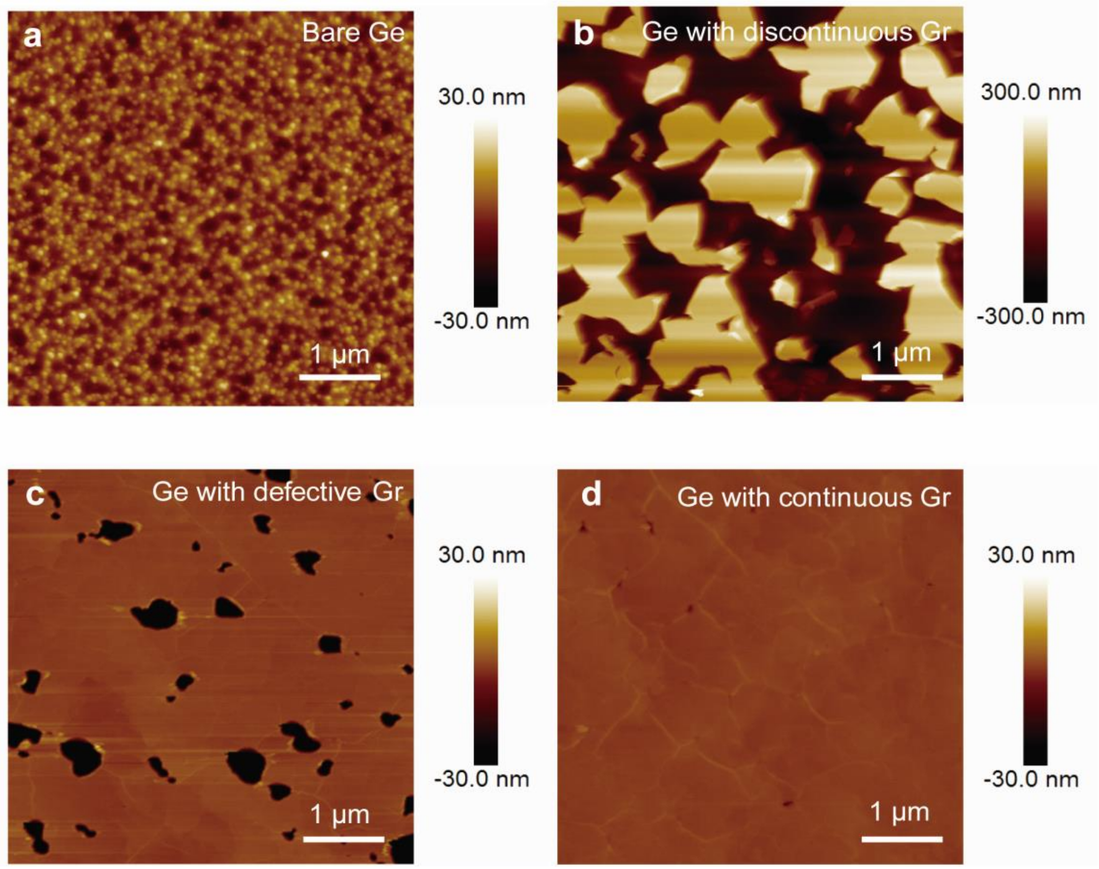

Supplementary Figure 1: Topology images of samples after the fluorination in SF_6 plasma for 180 s (a) bare Ge(111), (b) Ge(111) with discontinuous graphene coverage, (c) Ge(111) with defective graphene coverage, and (d) Ge(111) with continuous graphene coverage.

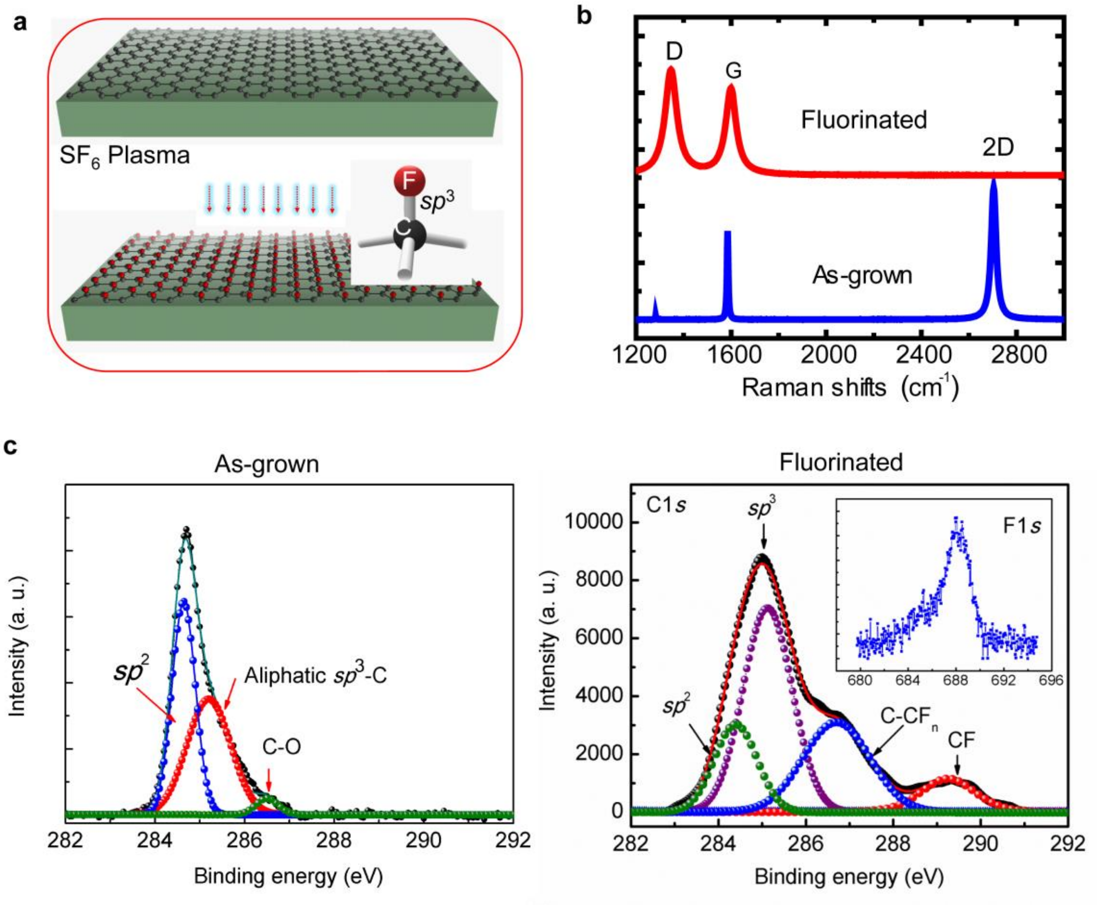

Supplementary Figure 2: Chemical characterization of fluorinated graphene. (a) Schematic diagram of single side fluorination process conducted on graphene/Ge (111). (b) Raman spectra of as-grown and fluorinated graphene. (c) XPS C1*s* spectra collected from as-grown and fluorinated graphene. The appearance of F1*s* in fluorinated graphene is exhibited as inserted.

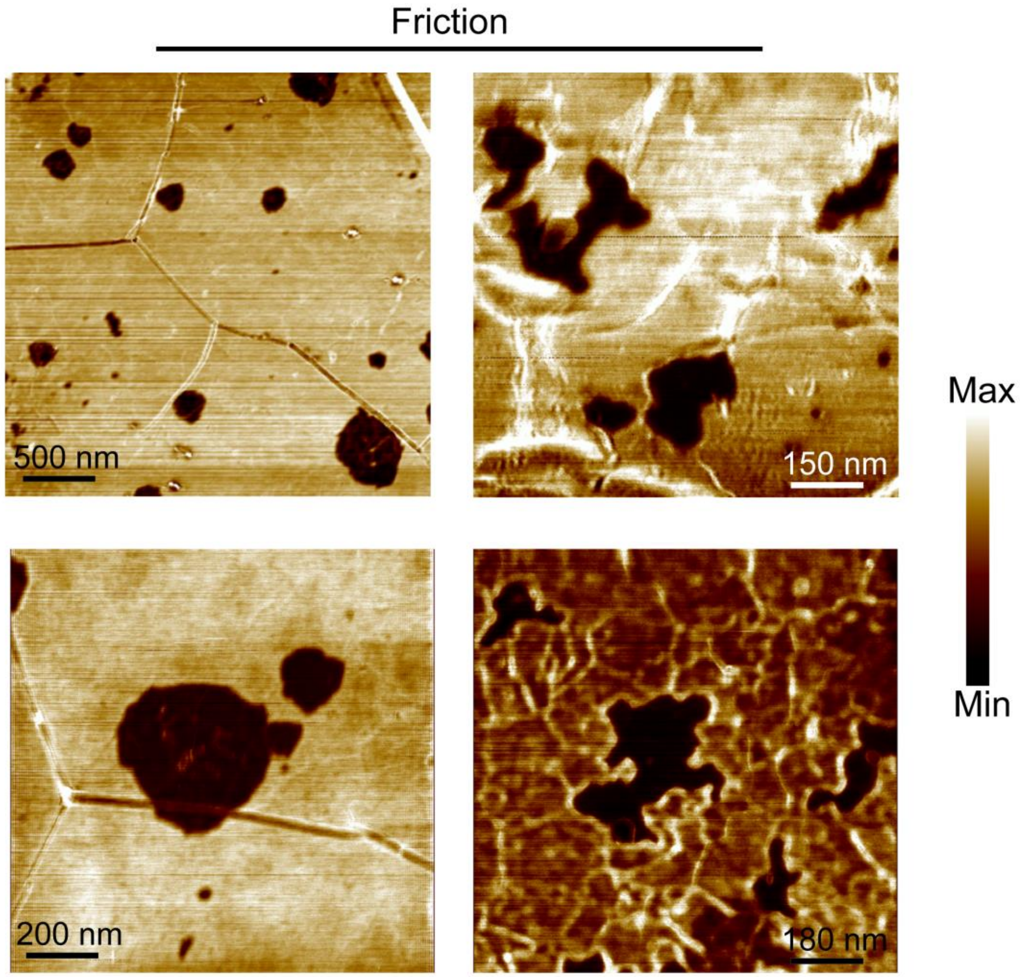

Supplementary Figure 3: Frictional behaviors of fluorinated graphene. Friction images are collected from various fluorinated graphene/Ge(111) heterostructures. The preservation of low friction islands embraced by high-friction bases can be observed in each fluorinated graphene/Ge(111) heterostructure.

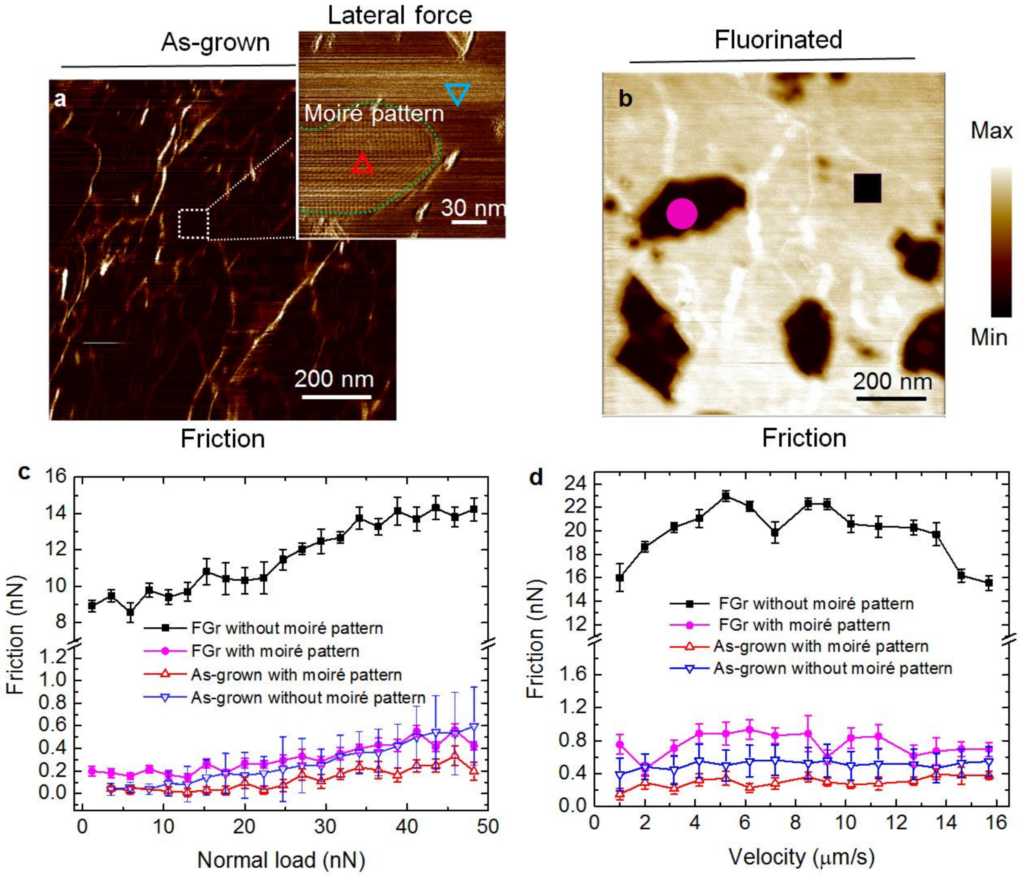

Supplementary Figure 4: Load and velocity dependences of friction. (a) Friction images acquired from as-grown graphene, a uniform low friction can be observed. Regions with/without moiré pattern can be distinguished in a zoom-in scan of the lateral force image inserted in figure 4a. (b) Friction image acquired from fluorinated graphene, islands with ultralow friction can be observed as discussed in the main text. (c) Friction signal gradually increases with the normal loads in regions with/without moiré pattern on samples of as-grown and fluorinated graphene. (d) Friction signals in regions with/without moiré pattern on samples of as-grown and fluorinated graphene fluctuate slightly when the scanning velocity increased from 1 μm/s to ~16 μm/s, nevertheless the contrast among them remained qualitatively similar.

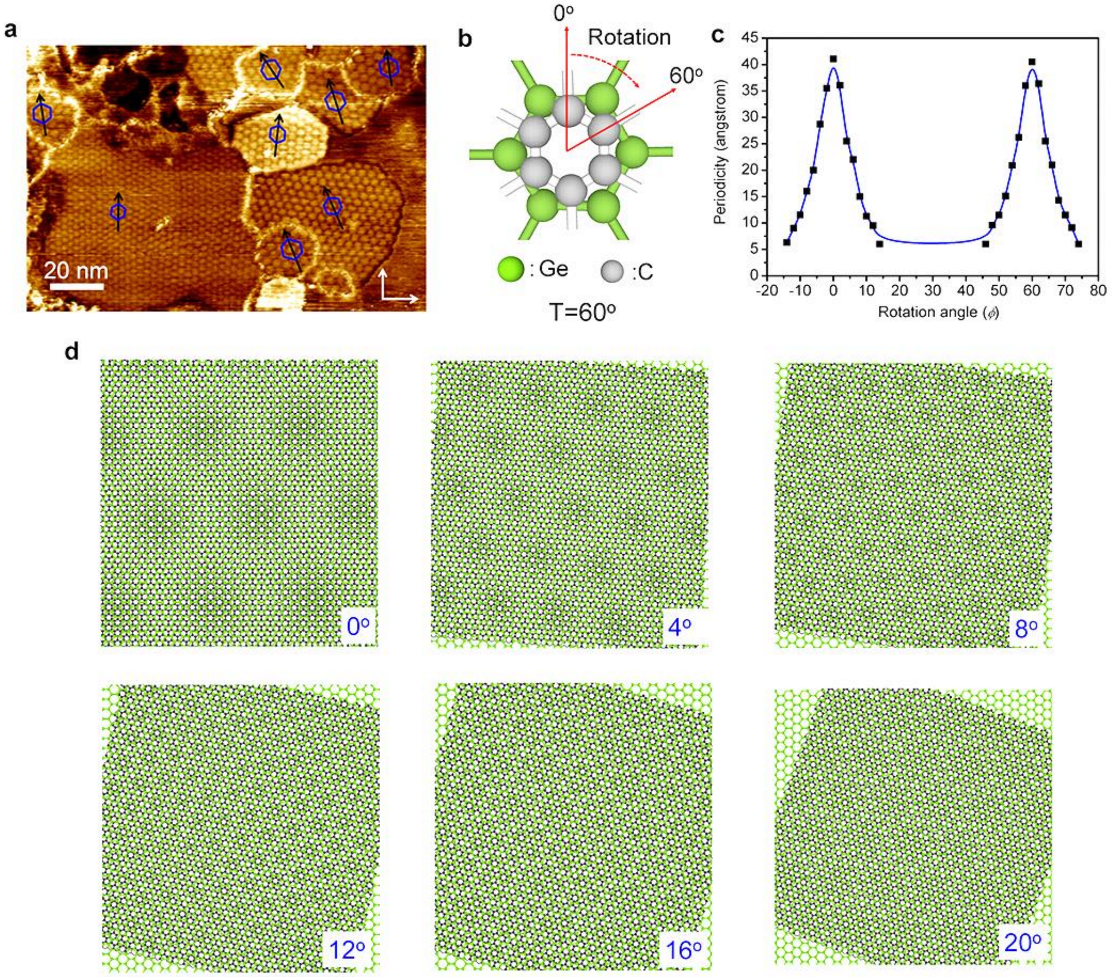

Supplementary Figure 5: Moiré pattern of graphene/Ge(111) heterostructure. (a) STM image shows that various moiré patterns with different periodical lengths and orientations can be formed between graphene and Ge(111), indicating the graphene grown on Ge(111) is polycrystalline. (b) Defining the rotation angle of graphene relative to Ge(111) crystal plane. (c) Periodical length of moiré pattern versus the rotation angle of graphene with respect to the Ge(111) crystal plane. (d) The morphologies of moiré patterns at rotation angles of 0°, 4°, 8°, 12°, 16° and 20°, respectively.

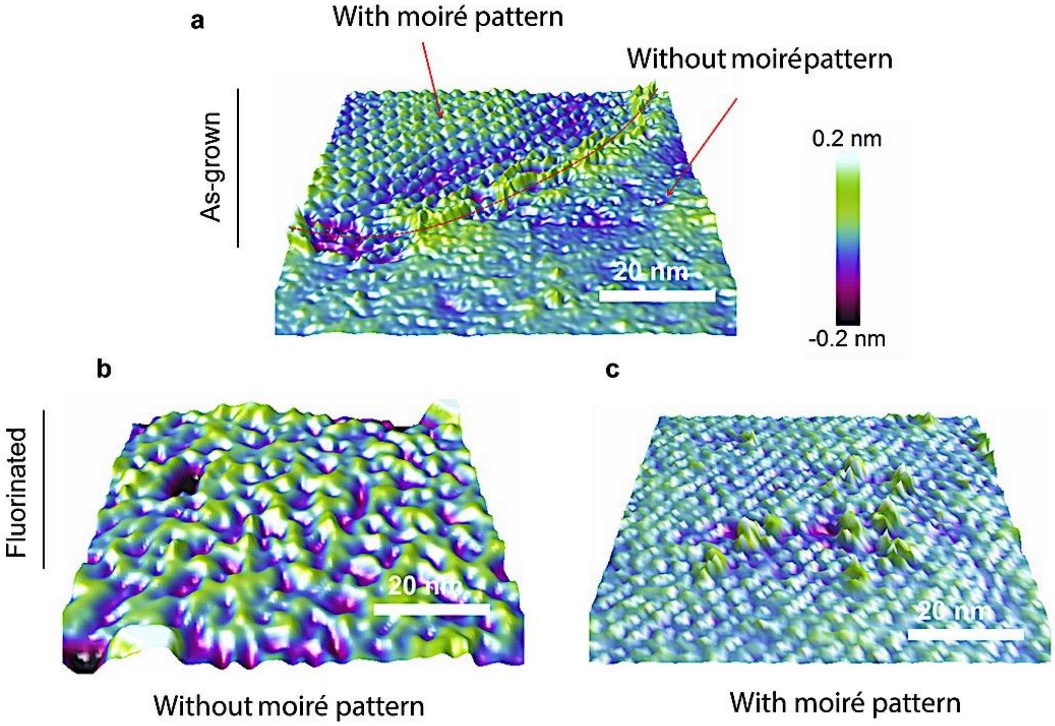

Supplementary Figure 6: Atomic-resolution STM topography of as-grown and fluorinated graphene. (a) STM topography of as-grown graphene on Ge(111) containing regions with/without moiré pattern. The surface roughness obtained from two regions of as-grown graphene are quite similar, except for the regular undulation of moiré pattern. (b,c) STM topography images obtained from the regions without and with moiré pattern of fluorinated graphene, respectively. For the fluorinated graphene without moiré pattern, the severe corrugation at molecule-level is observed. However, the corrugation phenomenon is absent on the moiré pattern region treated by the same fluorination process.

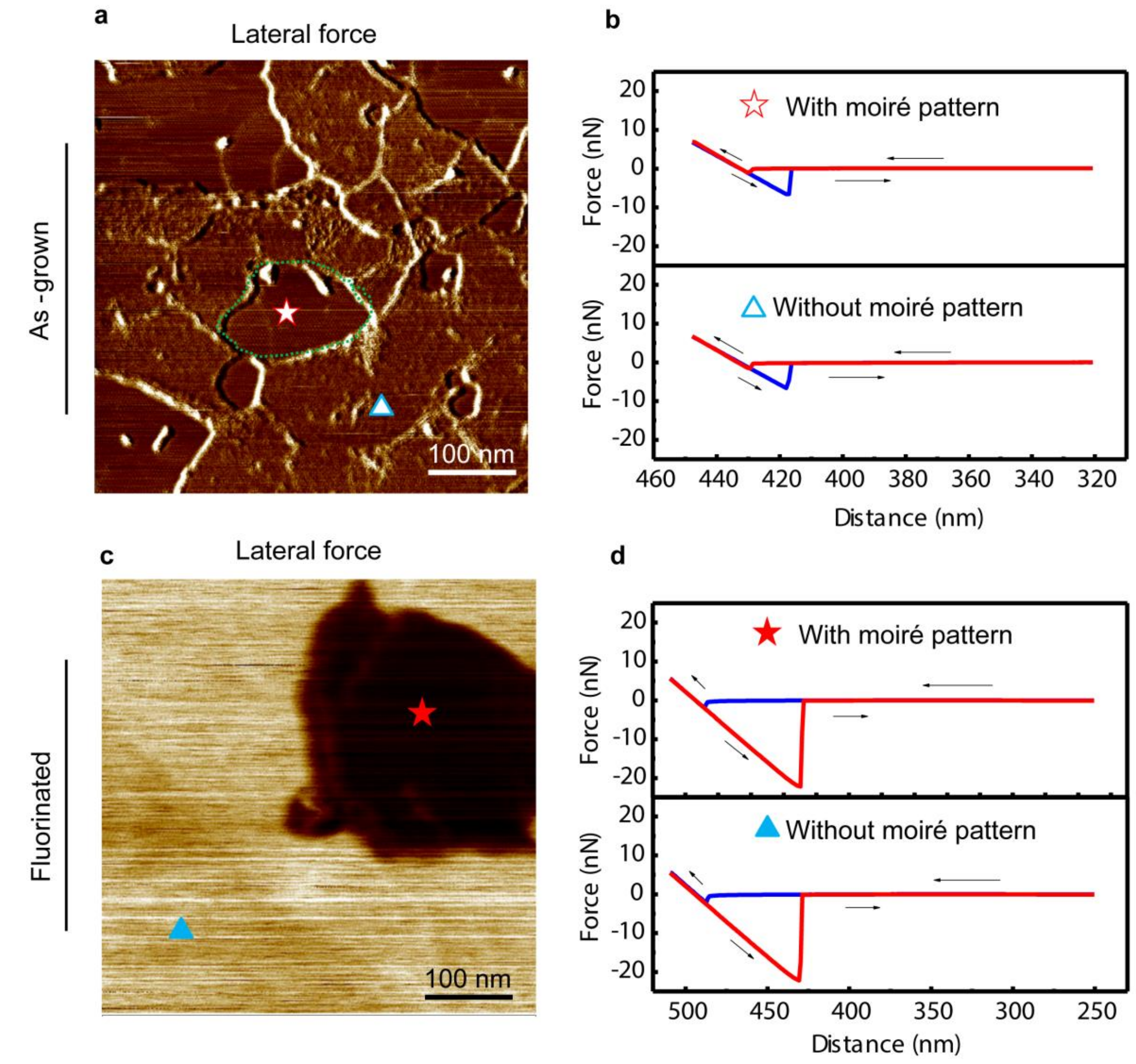

Supplementary Figure 7: Pull-off force measurements in atmospheric environment. (a,c) Lateral force images obtained from as-grown graphene and fluorinated graphene containing regions with/without moiré pattern. (b,d) Corresponding pull-off force curves acquired precisely on regions with/without moiré pattern as marked by star and triangle in figures 7a and 7c.

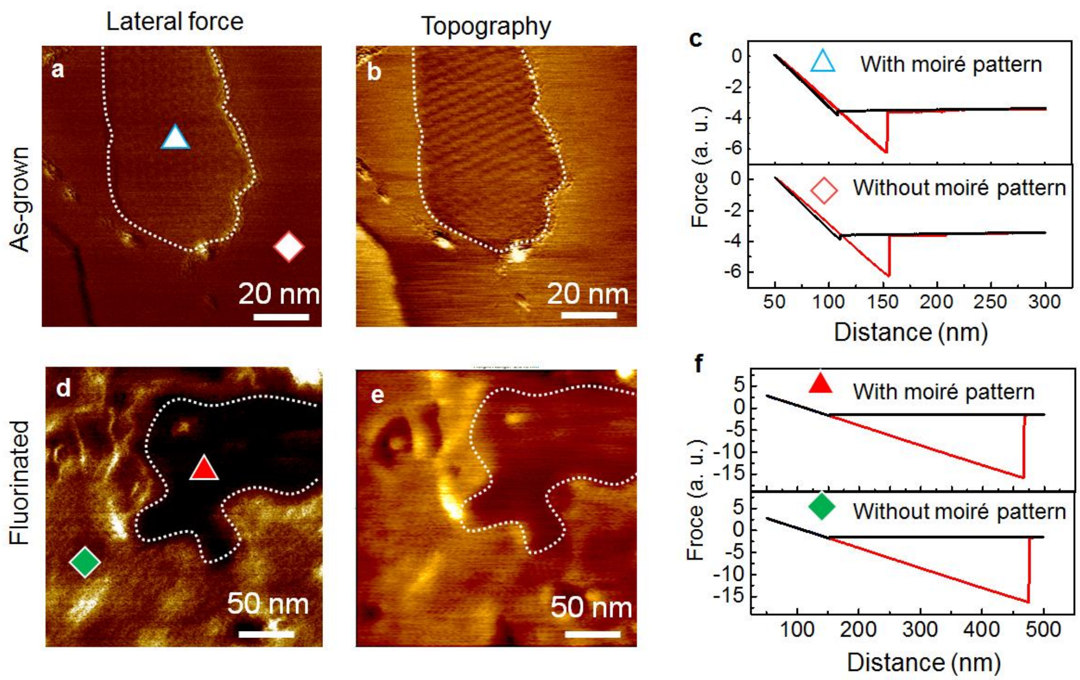

Supplementary Figure 8: Pull-off force measurements in dry N_2 environment. Lateral force and topography images obtained from (a,b) as-grown graphene and (d,e) fluorinated graphene containing regions with/without moiré pattern. (c,f) Corresponding pull-off force curves acquired precisely on regions with/without moiré pattern as marked in figures 8a and 8d. The measurements were carried out with a silicon nitride AFM tip in N_2 with the relative humidity of ~5% at room temperature.

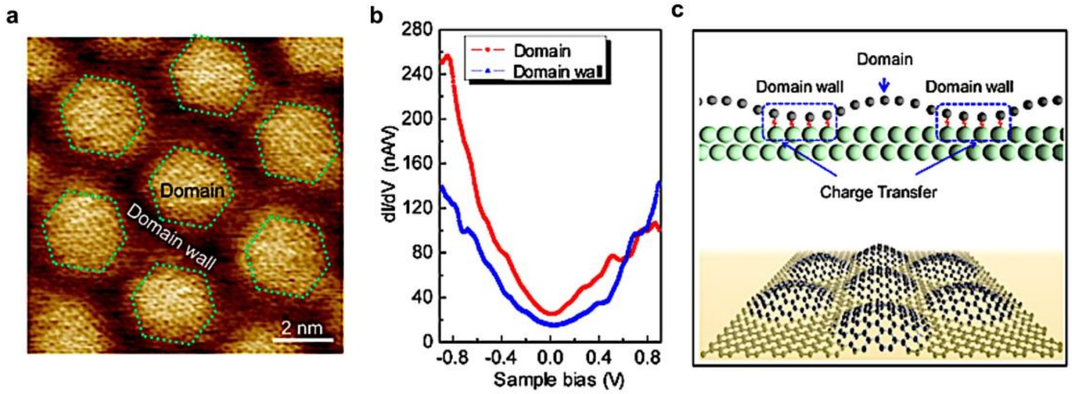

Supplementary Figure 9: Moiré pattern of graphene/Ge(111) heterostructure. (a) Atomic resolution STM image of moiré pattern region formed in graphene grown on Ge(111) substrate. It contains domain (bright) and domain wall (dark) regions in each unit. (b) d*I*/d*V* curves recorded at different spatial positions (bright (domain) and dark (domain wall). The difference indicates charge transfer occurs in the domain wall. (c) Schematically drawing of C and Ge atomic locations in the moiré pattern.

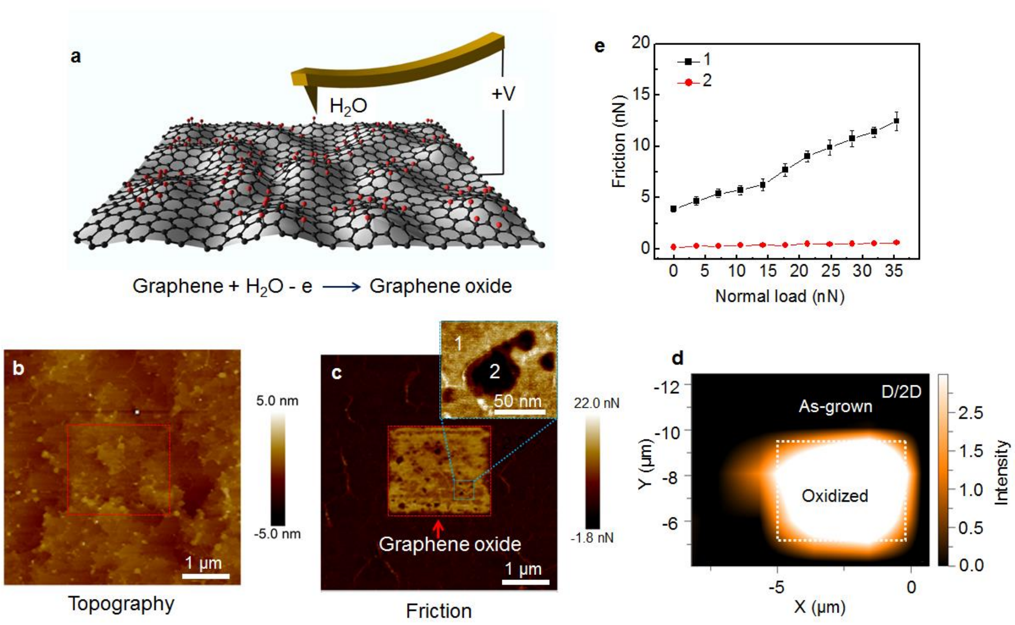

Supplementary Figure 10: Friction behavior of graphene on Ge(111) substrate oxidized by AFM tip lithography technology. (a) Schematic diagram of the AFM lithography setup for local oxidation of graphene. (b,c) Topography and friction images acquired from the oxidation region. $2 \times 2\ \mu m^2$ area of oxidized graphene can be observed in the friction panel, a low-friction island can be distinguished in the inserted zoom-in image. As observed in the fluorinated graphene, the base region with high friction and the island region with low friction coexist on oxidized graphene as well. (d) Raman mapping of the oxidized region showing the dramatic increment of D/2D peak ratio. (e) Plot of friction force versus applied load collected from base and island regions on oxidized graphene marked in the insert of figure 10c, respectively.

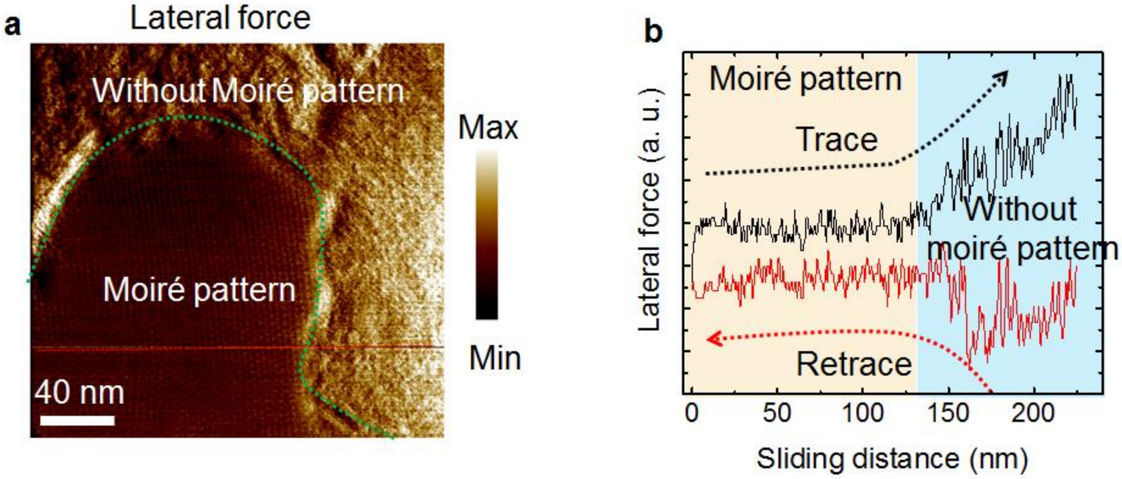

Supplementary Figure 11: High-resolution friction image of oxidized graphene. (a) High-resolution lateral force image of regions with/without moiré pattern on oxidized graphene sample. (b) Trace and re-trace scanning lines acquired from figure 11a along the red dashed line.

Supplementary Note 1: Characteristics of fluorinated graphene on Ge(111)

SF_6 plasma is a strong etching agent for Ge substrate but not so for graphene. In our experiments, we find that regions of the Ge surface covered by graphene film are generally protected from etching. Similar effects have been reported for graphene on Cu or Ni surfaces[1,2]. As shown in Supplementary Figure 1a, when pure Ge(111) is exposed to SF_6 plasma, the surface will be severely etched. When Ge(111) is covered by the discontinuous graphene, the exposed Ge surface will be etched as subjected to SF_6 plasma (Supplementary Figure 1b). If the covered graphene film is continuous but with some defects, F^- ions will only etch the Ge substrate underneath the defects of graphene (Supplementary Figure 1c). In this study, the graphene growth condition is fully optimized to achieve super quality graphene, so the SF_6 etching phenomenon can be totally precluded, as shown in Supplementary Figure 1d.

The changes induced by fluorination (Supplementary Figure 2a) have been corroborated by Raman spectroscopy and X-ray photoelectron spectroscopy (XPS). Raman scattering (HORIBA JobinYvon HR800) was conducted using an Ar^+ laser with a wavelength of 514 nm and a spot size of 1 μm. The spectra were recorded with a 600 lines/mm grating. The ESCALAB 250 XPS manufactured by THERMO VG SCIENTIFIC LTD was employed to study the chemical states of the surface with the monochromatic Al K_α x-ray source. All the measurements were

performed in an ultra-high-vacuum chamber with a base pressure of 10^{-10} Torr at room temperature. Fitting of the XPS spectra was performed using a Gussian-Lorentzian peak shape after performing Shirley background correction.

Raman spectrum collected from the as-grown graphene shows a G band at 1593 cm^{-1} and a 2D band at 2700 cm^{-1} (Supplementary Figure 2b). The 2D band exhibits a symmetric single Lorentzian line shape with a full-width half-maximum (FWHM) of 28.1 cm^{-1} and the intensity ratio of 2D to G bands is about 2.1, which corresponds to the features of monolayer graphene with good uniformity, as reported previously[3,4]. However, 2D peak almost disappears and G peak becomes broad after the fluorination (Supplementary Figure 2b). Meanwhile, the fluorination process also induces the appearance of sharp defect-related D band (~1350 cm^{-1}). The activation of D band demonstrates the breaking of the translational symmetry of C-C sp^2 bonds after the formation of sp^3 hybridized bonding of a carbon atom with a F atom[5]. Meanwhile, defects also perturb electronic states and decrease its life time, thus resulting in the decrease of 2D band[6]. The formation of sp^3 hybridized carbon-fluorine bonds by fluorination is further verified by comparison of C1*s* core-level spectra of XPS of the fluorinated graphene. XPS results of the as-grown and fluorinated graphene are presented in Supplementary Figure 2c. The major peak at 284.6 eV of C1*s* originates from graphene's non-functionalized sp^2 bonding C atoms, while the minor peaks located at 285.1 eV and 286.7 eV are attributed to aliphatic non-functionalized sp^3

C atoms and C-O bonding from the carbon contaminations[7,8]. In the fluorinated graphene, the enhancement of the peak at 285.1 eV (sp^3) together with the attenuation of the peak at 284.6 eV (sp^2) is associated with the distorted sp^2 bonding C atoms which have one neighboring C atom bonded with one F atom, i.e., the C-CF bonds[6,9,10]. In addition, the pronounced F1*s* peak locates at 688.5 eV (inset in Supplementary Figure 2c) and the extra component at 289 eV in the C1*s* peak also prove the existence of F atoms and F-C bonds[11,12]. The atomic fractions of carbon and fluorine atoms estimated from the XPS data are ~74% and ~26%, respectively. Properties of the fluorinated graphene have been described in our previous work[13].

Supplementary Note 2: Moiré pattern of graphene/Ge(111) heterostructure

For epitaxially grown graphene on Ge(111), lattice mismatch between graphene and Ge(111) is fixed (~39.5%). The orientation and periodical length of the moiré pattern are determined by the relative rotation angle between graphene and the underneath Ge(111) substrate[14]. Occasionally, moiré patterns with multiple orientations can be observed in a same region (200 nm×150 nm), as shown in Supplementary Figure 5a, which directly confirms that graphene grown on Ge(111) is polycrystalline with a weak domain orientation preference. Based on the lattice parameters, we schematically constructed a series of heterostructures, each consisting of one layer of graphene and one layer (containing two sub-layers) of Ge(111), to demonstrate the dependence of the period of moiré pattern on the relative rotation angle. We have defined the aligned case (0°) and the rotation direction as schematically shown in Supplementary Figure 5b. It can be deduced that the period of the rotation (T) is 60°. Supplementary Figure 5d depicts the formation of moiré patterns at rotation angles of 0°, 4°, 8°, 12°, 16° and 20°, respectively. The periodical length of the moiré pattern as a function of the relative rotation angle has been summarized in Supplementary Figure 5c. It can be observed visible moiré pattern appears in the small range of rotation angle, which is denoted as nearly aligned case. Periodical length reaches the maximum value of ~4 nm when graphene is well aligned (0°) with the underneath Ge, then

decreases rapidly as the rotation angle increases. Moiré pattern becomes almost invisible when graphene is highly misaligned on Ge(111) which is denoted misaligned case. The results are in accordance with the high resolution friction and STM results.

Supplementary Note 3: Adhesion measurements on as-grown and fluorinated graphene

Pull-off force curves were obtained from the regions with/without moiré pattern on as-grown graphene and fluorinated graphene, as shown in Supplementary Figure 7. For comparison, the characterization conditions and the scanning tip were kept identical during the whole measurement. As depicted in Supplementary Figure 7a, regions with/without moiré pattern can be distinguished in the friction image of as-grown graphene. After repeated measurements, no difference in the pull-off force curves is observed between two representative regions (as marked by star and triangle) which suggests the corresponding adhesion properties are similar, as shown in Supplementary Figure 7b. The detach force is about 6 nN for the two regions which indicates the weak stickiness in the as-grown graphene. When the similar measurement is carried out on fluorinated graphene as shown in Supplementary Figure 7c, the detach forces from the regions with/without moiré pattern are both enhanced significantly (more than 20 nN) (Supplementary Figure 7d) due to the formation of complex C_nF structures. However, the detach force from the moiré pattern region of fluorinated graphene is still similar to that from non-moiré pattern region (Supplementary Figure 7d), suggesting both regions have the similar fluorination behaviors.

To rule out the possible influence of capillary, we also carried out pull-off force measurements in dry N_2 environment (relative humidity around 5%) at room temperature. As

shown in Supplementary Figures 8a and 8b, the moiré pattern can be observed in lateral force and topography images in the as-grown graphene/Ge(111). We performed the pull-off force measurements on two regions with and without moiré pattern using a SiN tip. As shown in Supplementary Figure 8c, no significant difference in the pull-off force curves is observed between these two representative regions (as marked by square and triangle). For the fluorinated graphene (Supplementary Figures 8d and 8e), the pull-off force increases noticeably compared to the as-grown sample in both regions with/without moiré pattern (Supplementary Figure 8f). However, there is still no significant difference in pull-off force between moiré and non-moiré regions. The data obtained from both Supplementary Figures 7 and 8 suggest that the friction contrast between the region with moiré pattern and the region without moiré pattern always exists for different measurement environments. In addition, the pull-off force is closely related to the surface chemical state, therefore, the possibility for less fluorination in the moiré pattern regions can be precluded, as discussed in the main text.

Supplementary Note 4: Morphology and frictional behaviors of oxidized graphene

Besides the fluorinated graphene, the frictional behavior of oxidized graphene/Ge(111) heterostructure has been studied as well. Oxidation of graphene was obtained directly through tip-lithography method. It was performed at ambient conditions (temperature of 24 ^{o}C and relative humidity of ~ 40%) by a contact mode AFM (Multimode 8 SPM system) with a Pt/Ir coated conductive tip (DPE-XSC11, MIKROMASCH). During scanning, a local dc bias voltage of +12 V between the AFM tip and the graphene sample was scanned over the region of 2×2 μm^2. The oxidation process was implemented by tip assisted electrochemical effects as reported previously[15]. Supplementary Figure 10a shows a schematic diagram of the AFM lithography setup for local oxidation of graphene. When a positive dc bias voltage (high enough) is applied on the conducted AFM tip, it is expected to decompose water molecules adsorbed on graphene in the ambient environment into ions (H^+, OH^-, and O^{2-}). Then, the tip acts as the anode to assist the oxidation of the underneath graphene[15]. Even though the oxidation seems to introduce negligible change in the surface morphology (Supplementary Figure 10b), the surface friction increases dramatically on the oxidized region, as shown in Supplementary Figure 10c. The observations are in accordance with the previous reports[15-18]. Zoom in the friction image of oxidized graphene (insert in Supplementary Figure 10c), there are clear islands with low friction as that on the

fluorinated samples. Raman mapping on the oxidized region (Supplementary Figure 10d) shows the tip-assisted oxidation induces the appearance of sharp defect-related D band with a decaying 2D band, which suggests the breaking of the translational symmetry of C-C sp^2 bonds. We have also measured the friction behaviors versus the normal applied load on the oxidized graphene (Supplementary Figure 10e). Friction in the base region without moiré pattern increases significantly as the applied load increases, however, the island with moiré pattern always keep their low friction state, which is also observed in fluorinated graphene (Fig. 1f in the main text).

By taking a high-resolution friction image on the oxidized region (Supplementary Figure 11a), we confirmed that the ultra-low friction island region indeed exhibited a moiré pattern, while the moiré pattern was absent in the base region with high friction (Supplementary Figure 11b). These results support our hypothesis that the mechanism of ultra-low friction state preservation for the fluorinated graphene is qualitatively similar to that for the oxidized graphene.

Supplementary References

1 Chen, S. *et al.* Oxidation resistance of graphene-coated Cu and Cu/Ni alloy. *ACS Nano* **5**, 1321-1327, (2011).

2 Robinson, J. *et al.* Properties of fluorinated graphene films. *Nano Lett.* **10**, 3001-3005, (2010).

3 Wang, G. *et al.* Direct growth of graphene film on germanium substrate. *Sci. Rep.* **3**, 2465, (2013).

4 Lee, J.-H. *et al.* Wafer-scale growth of single-crystal monolayer graphene on reusable hydrogen-terminated germanium. *Science*, **344**, 286-289, (2014).

5 Malard, L. M., Pimenta, M. A., Dresselhaus, G. & Dresselhaus, M. S. Raman spectroscopy in graphene. *Phys. Rep.* **473**, 51-87, (2009).

6 Tahara, K., Iwasaki, T., Matsutani, A. & Hatano, M. Effect of radical fluorination on mono- and bi-layer graphene in Ar/F_2 plasma. *Appl. Phys. Lett.* **101**, 163105, (2012).

7 Díaz, J., Paolicelli, G., Ferrer, S. & Comin, F. Separation of the sp^3 and sp^2 components in the C1s photoemission spectra of amorphous carbon films. *Phys. Rev. B* **54**, 8064-8069, (1996).

8 Han, N. *et al.* Improved heat dissipation in gallium nitride light-emitting diodes with embedded graphene oxide pattern. *Nat. Commun.* **4**, 1452, (2013).

9 Dimiev, A. *et al.* Layer-by-layer removal of graphene for device patterning. *Science* **331**, 1168-1172, (2011).

10 Sahin, H., Topsakal, M. & Ciraci, S. Structures of fluorinated graphene and their signatures. *Phys. Rev. B* **83**, 115432, (2011).

11 Shulga, Y. M. *et al.* XPS study of fluorinated carbon multi-walled nanotubes. *J. Electron Spectrosc.* **160**, 22-28, (2007).

12 Kwon, S. *et al.* Enhanced nanoscale friction on fluorinated graphene. *Nano Lett.* **12**, 6043-6048, (2012).

13 Zheng, X. *et al.* Fluorinated graphene in interface engineering of Ge-based nanoelectronics. *Adv. Funct. Mater.* **25**, 1805-1813, (2015).

14 Wang, B. *et al.* Coupling epitaxy, chemical bonding, and work function at the local scale in transition metal-supported graphene. *ACS Nano* **4**, 5773-5782, (2010).

15 Byun, I.-S. *et al.* Nanoscale lithography on monolayer graphene using hydrogenation and oxidation. *ACS Nano* **5**, 6417-6424, (2011).

16 Ding, Y. H. *et al.* Intrinsic structure and friction properties of graphene and graphene oxide nanosheets studied by scanning probe microscopy. *B. Mater. Sci.* **36**, 1073-1077, (2013).

17 An, Y. F. *et al.* Friction and wear properties of graphene oxide/ultrahigh-molecular-weight polyethylene composites under the lubrication of deionized water and normal saline solution. *J. Appl. Polym. Sci.* **131**, 39640, (2014).

18 Dong, Y. L., Wu, X. W. & Martini, A. Atomic roughness enhanced friction on hydrogenated graphene. *Nanotechnology* **24**, 375701, (2013).
